# Perchlorates on Mars enhance the bacteriocidal effects of UV light

**DOI:** 10.1038/s41598-017-04910-3

**Published:** 2017-07-06

**Authors:** Jennifer Wadsworth, Charles S. Cockell

**Affiliations:** UK Centre for Astrobiology, School of Physics and Astronomy, University of Edinburgh, Edinburgh, EH10 4EP UK

## Abstract

Perchlorates have been identified on the surface of Mars. This has prompted speculation of what their influence would be on habitability. We show that when irradiated with a simulated Martian UV flux, perchlorates become bacteriocidal. At concentrations associated with Martian surface regolith, vegetative cells of *Bacillus subtilis* in Martian analogue environments lost viability within minutes. Two other components of the Martian surface, iron oxides and hydrogen peroxide, act in synergy with irradiated perchlorates to cause a 10.8-fold increase in cell death when compared to cells exposed to UV radiation after 60 seconds of exposure. These data show that the combined effects of at least three components of the Martian surface, activated by surface photochemistry, render the present-day surface more uninhabitable than previously thought, and demonstrate the low probability of survival of biological contaminants released from robotic and human exploration missions.

## Introduction

Perchlorates have been detected on Mars both *in-situ* ^1^ and inferred in brine seeps^2^, which raises questions on their effects on the habitability of that planet. The implications are significant as their detection suggests the presence of other oxychlorine species, which may negatively impact the habitability of Mars and interfere with the preservation and detection of organic material^3^. Conversely, the presence of such salts lowers the freezing point of water^4, 5^ thereby potentially allowing for a contemporary active hydrological system on Mars, which could enhance the habitability of the near-surface environment. This has prompted recent research into the use of perchlorates as a potential energy source for bacteria on Mars^6, 7^. Consisting of a negatively charged chloride surrounded by a tetrahedral formation of oxygen atoms, perchlorates represent the highest oxidation state of chlorine (+7) and are powerful oxidants when heated, but are stable at room temperature and lower temperatures. In biology, the high oxidation state of perchlorates means that they can be used as an electron acceptor by microorganisms to provide energy for growth^8^. The presence of oxidants in the Martian soil was first suspected during the Viking Lander missions^9, 10^. The missions suggested low levels of reactive oxidizing substances, which were thought to explain why no evidence for organics was found^11, 12^. The detection of chloro-hydrocarbons was initially considered to be terrestrial contamination but could be explained by the presence of oxychlorine species upon re-analysis^13^. In 2008, the NASA Phoenix Lander’s onboard Wet Chemistry Lab eventually discovered the presence of perchlorate anions, at a concentration of 0.4–0.6 wt%^1^. This finding was recently supported by the Sample Analysis at Mars instrument (SAM) on the Curiosity rover^14^. In September 2015, the Mars Reconnaissance Orbiter spectroscopically detected hydrated salts of NaClO_4_, Mg(ClO_4_)_2_ and Mg(ClO_3_)_2_ in locations thought to be brine seeps. This may be the first direct evidence for flowing liquid water containing hydrated salts on Mars^2^.

Despite the stability and lack of reactivity of perchlorate at ambient temperatures, once heated, it becomes a well-characterized and highly effective oxidizing agent^15^, used as solid rocket fuel^16^. We investigated its potential reactivity by irradiating perchlorates under UV and observed its effect on the viability of a model vegetative organism, *Bacillus subtilis*, which is a common spacecraft contaminant^17, 18^. We report the significant bacteriocidal effects of UV-irradiated perchlorate on life at ambient temperatures and under Martian conditions.

## Results

### Bacteriocidal effect of UV-irradiated perchlorate

To determine if perchlorate had an effect on cell viability, *Bacillus subtilis* cells in minimal media M9 were irradiated in the presence of dissolved magnesium perchlorate (Mg(ClO_4_)_2_) at a concentration (0.6 wt%) typical of the Martian surface. Magnesium perchlorates were used as perchlorate have been detected in Martian soils directly^1^, are a putative component of brine seeps and magnesium perchlorate is thought to be a specific component^2^. However, as it is in solution, we are solely interested in the perchlorate ion and its affects of cell viability. Experiments were conducted under a monochromatic UV radiation source at 254 nm. Mars is subjected to UVC (200–280 nm) radiation on account of the lack of a significant oxygen concentration or ozone shield and a lower cut-off caused by CO_2_. The flux of 254 nm radiation we chose was similar to the absolute flux of radiation between 200 and 315 nm (UVC and UVB radiation), the most damaging region of the UV radiation spectrum to DNA^19^. We quantified the harmful effect on viability by calculating the ratio of surviving cells (‘N’) with regard to the starting concentration (‘N_0_’). We defined ‘viability’ as any number of cells greater than zero, consequently ‘sterility’ as zero cells. Results are shown on a log scale in all figures, therefore cases that are strictly zero are not represented on the log plot but should be interpreted as strictly zero. Statistical significance was defined as p < 0.05. Numerical results are summarized in Supplementary Table S1.

**Figure 1:**
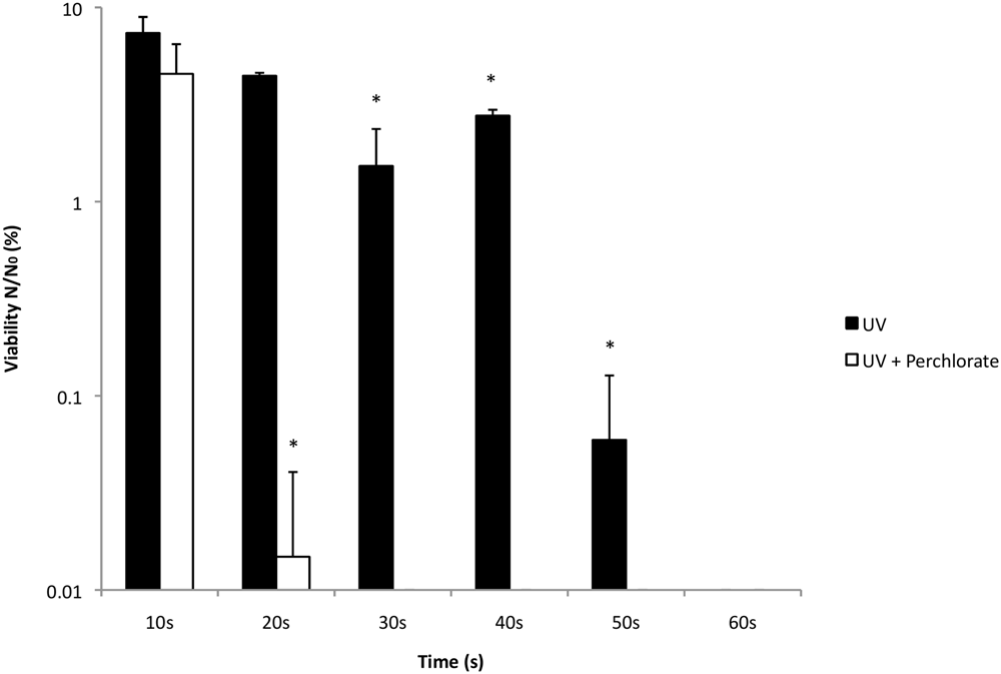
Effects of UVC-irradiated Mg(ClO_4_)_2_ on *B. subtilis* viability. UV = UVC irradiated control; Perchlorate = 0.6 wt% Mg(ClO_4_)_2_. p < 0.05 was considered statistically significant (*) among groups; error bars are + s.d. (n = 3).

Irradiated perchlorate had a significant bacteriocidal effect (Fig. 1). Cell viability was completely lost after 30 seconds exposure. By contrast, the control cells exposed to UV radiation without perchlorate took 60 seconds to be completely sterilized. Non-irradiated controls consisting of cells in M9, and cells in M9 in the presence of 0.6 wt% perchlorate, showed no significant difference in viability when left up to an hour (Supplementary Fig. S1).

### Bacteriocidal effect under Martian analogue conditions

**Figure 2:**
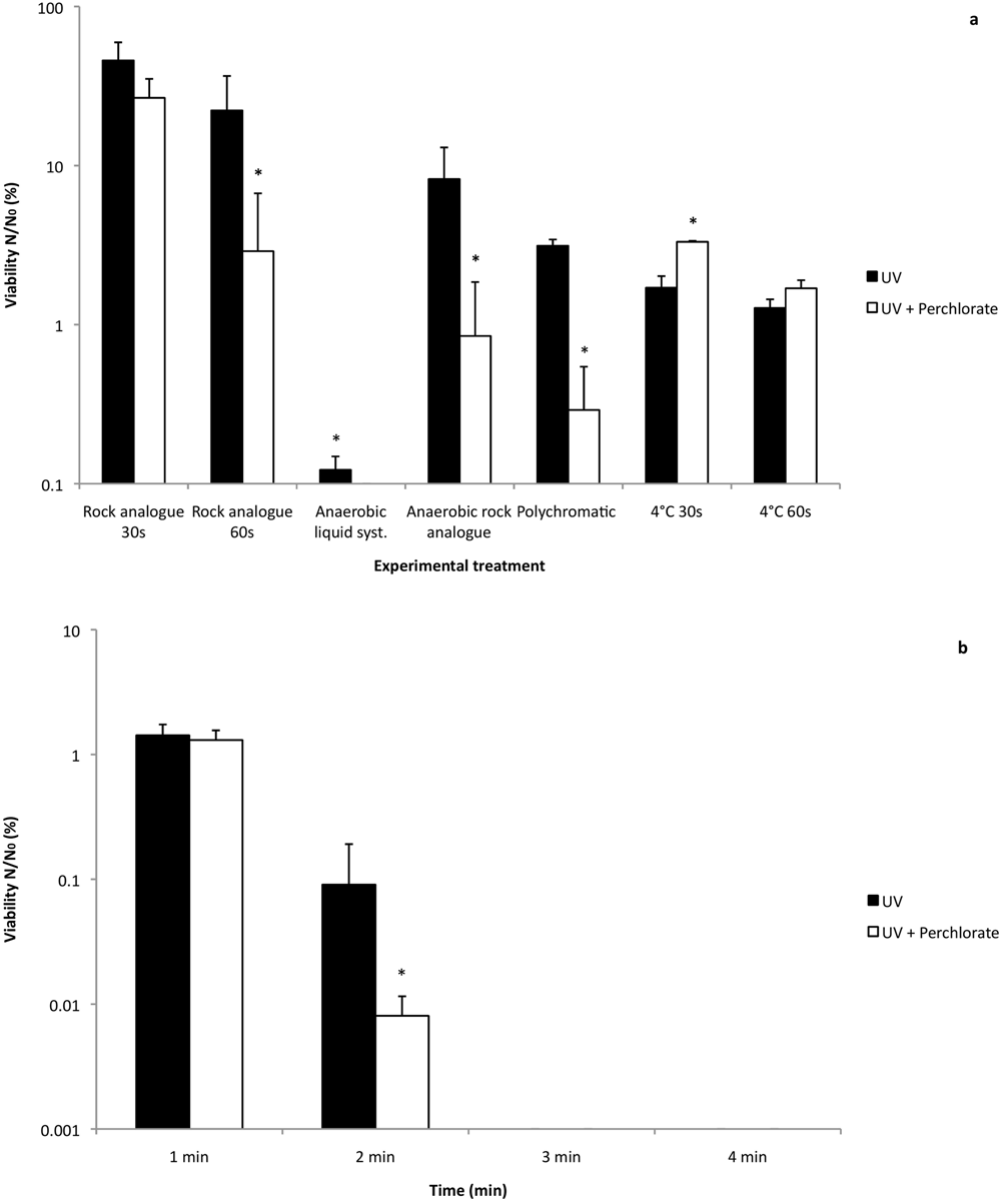
(**a**) Effects of UVC-irradiated Mg(ClO_4_)_2_ in rock analogues, under anaerobic conditions, polychromatic light and low temperature. Rock analogues exposed to aerobic environment (30 & 60 seconds);Liquid and rock analogue exposed to anaerobic environment (60 s); Liquid system exposed to polychromatic light (10 s); Liquid system chilled to 4 °C whilst irradiated (30 & 60 seconds); UV = UVC irradiated control; Perchlorate = 0.6 wt% Mg(ClO_4_)_2_. p < 0.05 was considered statistically significant (*) among groups; error bars are + s.d. (n = 3). (**b**) Effects of UVC-irradiated Mg(ClO_4_)_2_ at low temperature, 1–4 minute exposure. Liquid system chilled to 4 °C whilst irradiated. UV = UVC irradiated control; Perchlorate = 0.6 wt% Mg(ClO_4_)_2_. p < 0.05 was considered statistically significant (*) between or among groups; error bars are + s.d. (n = 3).

Although we showed irradiated perchlorates are bacteriocidal to cells in liquid media, we performed the same experiment under a number of Martian analogue conditions to test if this result was reproducible in a more representative environment. To simulate a rocky Martian habitat, the experiment was carried out using a simple system to more accurately simulate a rock environment (‘rock analogue system’) in which cells were deposited within silica discs. Although the overall cell survival was higher, Fig. 2a shows a significant 9.1-fold drop in viability in the irradiated perchlorate-treated samples after 60 seconds, whilst the UV-irradiated controls show a 2-fold viability decrease after the same exposure time.

We then carried out experiments to investigate whether the perchlorate-sterilization effect would still be observed under the influence of other environmental parameters relevant to Mars, namely anaerobic conditions, polychromatic irradiation and low temperature, the results of which are also shown in Fig. 2a. Firstly, the liquid system and rock analogue systems were irradiated under anaerobic conditions. Both systems showed that perchlorate-containing samples irradiated with UV experienced a greater loss of viability than the UV-irradiated controls. In the liquid system, cells remained viable (0.12%) after 60 seconds under just UV irradiation, but in the presence of perchlorate, viability was completely lost after 60 seconds. By contrast, in the rock analogue system, under UV irradiation greater cell viability was retained after 60 seconds (8.23%), but irradiated perchlorate still caused a significant loss of cell viability (9.7-fold decrease) compared to the UV-irradiated only control after 60 seconds.

We carried out experiments in the liquid system using polychromatic light to more accurately simulate a natural light spectrum. Under polychromatic light, cells in the presence of perchlorate showed a significant 10.8-fold decrease in viability compared to the polychromatic UV-irradiated only controls. Supplementary Fig. S2 shows the total UV irradiance on Mars compared to the measured absolute UV irradiance in the Mars chamber from 200–400 nm.

We observed an effect of low temperature on the reaction. When the experimental system was chilled to 4 °C before and during monochromatic irradiation we observed after 60 seconds that the cell viability of the UV-irradiated perchlorate samples did not drop significantly below that of the UV-irradiated controls (Fig. 2a). To test whether this was because UV-irradiated perchlorates were no longer effective at low temperatures or whether the chemical reaction was delayed, we performed the same experiment for 10 minutes, measuring at each minute (Fig. 2b shows minutes 1–4). Once again, after one minute of UV exposure both samples displayed no significant differences in viability. However, after two minutes of irradiation the irradiated perchlorate-treated samples showed a significant 11.4-fold decrease in viability in comparison to the UV controls; both UV and perchlorate samples were sterile after three minutes. It is unclear if low temperature reduced perchlorate activation, reduced the diffusion of photoproducts to the cells or reduced the rate of cellular damage. Nevertheless, even at low temperatures, irradiated perchlorates proved bacteriocidal.

To confirm the production of potential biologically damaging photoproducts during perchlorate irradiation, the absorption spectrum of an irradiated solution of perchlorate was measured in the UV radiation range. Potential photoproducts produced during irradiation could be hypochlorite (absorbance maximum = 290 nm), chlorite (absorbance maximum = 260 nm) and chlorine dioxide (absorbance maximum = 360 nm). We observed an increase in absorbance at 260 nm and to a slightly lesser extent at 290 nm. There was a negligible increase in absorbance at 360 nm (Supplementary Fig. S3). A control sample containing non-irradiated perchlorate was also measured at the same time points and no increase in absorbance at any of the wavelengths was observed.

To determine whether altering the concentration of perchlorate affected the loss of cell viability when irradiated, the 0.6 wt% perchlorate solution was serially diluted to yield 0.06 wt% and 0.006 wt% solutions, which were irradiated in the presence of cells. Supplementary Fig. S4 shows that 0.06 wt% and 0.006 wt% there were no statistically significant differences in cell viability compared to the UV-irradiated controls.

**Figure 3:**
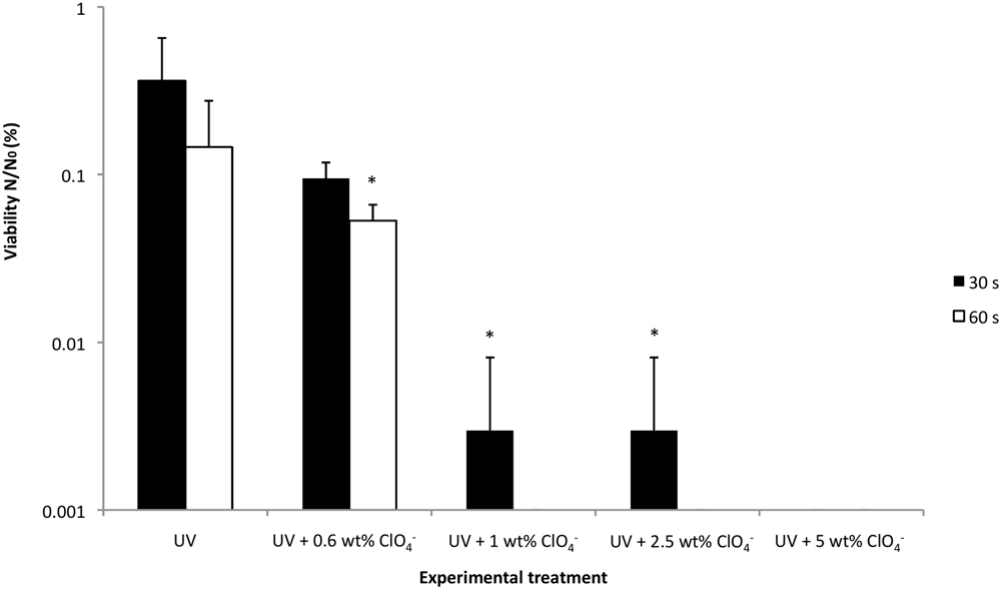
Influence of increased perchlorate concentration on bacteriocidal effects under UV irradiation. Mg(ClO_4_)_2_ at representative measured Martian concentration (0.6 wt %,), 1, 2.5 and 5 wt% (30 & 60 s); UV = UVC irradiated control (30 & 60 s). p < 0.05 was considered statistically significant (*) between and among groups; error bars are + s.d. (n = 3).

The effects of perchlorate were also investigated at higher concentrations than those measured in the Martian surface regolith (Fig. 3). Although the regolith contains a concentration of 0.4–0.6 wt%^1^, the spectral detection of putative perchlorate brines suggests that, in some local regions on Mars, the concentrations of this chemical could be much higher. At a perchlorate concentration of 1 wt% viability dropped over an order of magnitude after 30 seconds irradiation compared to results at 0.6 wt%. A complete loss of viability was observed after 60 seconds exposure. An increase of perchlorate concentration to 5 wt% resulted in complete loss of viability after only 30 seconds of irradiation.

### Interactions of other Martian soil components

After simulating the physical effects of the Martian environment on perchlorate activity, we also considered the additional soil components present and their potential interactions.

**Figure 4:**
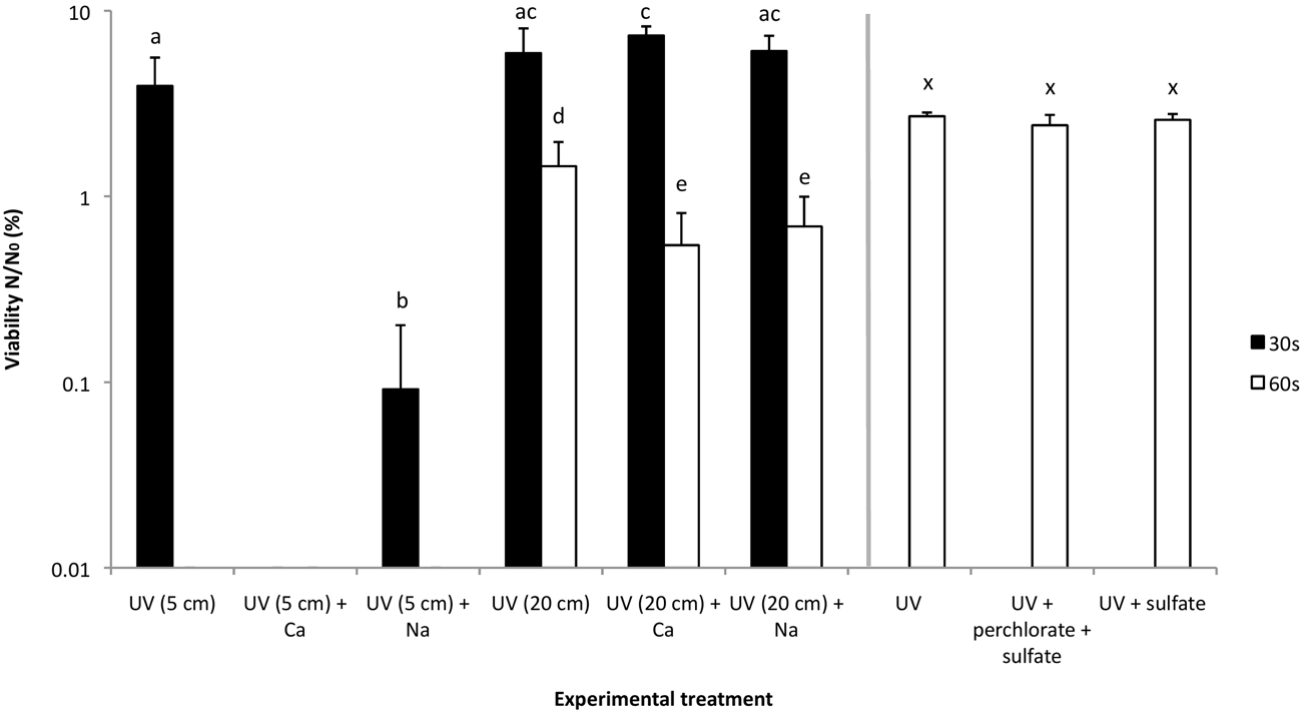
Effects of UVC-irradiated sodium/calcium perchlorate and sulfate on *B. subtilis* viability. UV = UVC irradiated control at given distance from light source; Ca = 0.6 wt% Ca(ClO_4_)_2_ at given distance from light source; Na = 0.6 wt% NaClO_4_ at given distance from light source; perchlorate = 0.6 wt % Mg(ClO_4_)_2_; sulfate = 30 wt% MgSO_4_. Letters shared in common between or among the groups indicate no significant difference (p > 0.05); error bars are + s.d. (n = 3). Vertical grey line indicates separate experiment with different control.

We undertook experiments to study whether other components of the Martian surface could affect the reactions we observed. Sulfate is an abundant component of the Martian regolith with ∼30 wt% of sulfate within sediments having been reported^20^. The experimental set up using perchlorate at 0.6 wt% was repeated but with the addition of sulfate at the estimated Martian concentration of 30 wt% to the perchlorate at 0.6 wt% (Fig. 4). The results show that there was no significant effect of sulfate on the loss of viability of the cells in the presence of UV-irradiated perchlorate, nor irradiated sulfate on its own did not differ significantly from the UV-irradiated control in terms of effects on viability.

We examined two additional forms of perchlorate that have been detected on Mars (Fig. 4). Sodium perchlorate (NaClO_4_) was detected in the reoccurring slope lineae by Ojha L. *et al*.^2^ and calcium perchlorate (Ca(ClO_4_)_2_) is thought to be the best candidate for the oxychlorine compounds found in Rocknest^14^.

The perchlorates were both irradiated with UVC at a concentration of 0.6 wt% for comparison with the magnesium perchlorate. The calcium and sodium perchlorates showed a significantly lower cell count than the UV-irradiated controls after 30 seconds. The calcium perchlorate-treated samples were completely sterilized and the sodium perchlorate-treated samples showed a 15-fold drop in viability compared to the controls; all samples were sterilized after 60 seconds UV radiation exposure. To get a better resolution of the effect of the perchlorates, they were additionally irradiated in the same set up at a four times greater distance from the light source (16 times less irradiance). These results showed no significant difference in viability in any samples in the first 30 seconds of irradiation. However, after 60 seconds both calcium and sodium perchlorate-treated samples showed significantly lower cell counts than the UV-irradiated controls (1.9 and 1.7-fold respectively).

**Figure 5:**
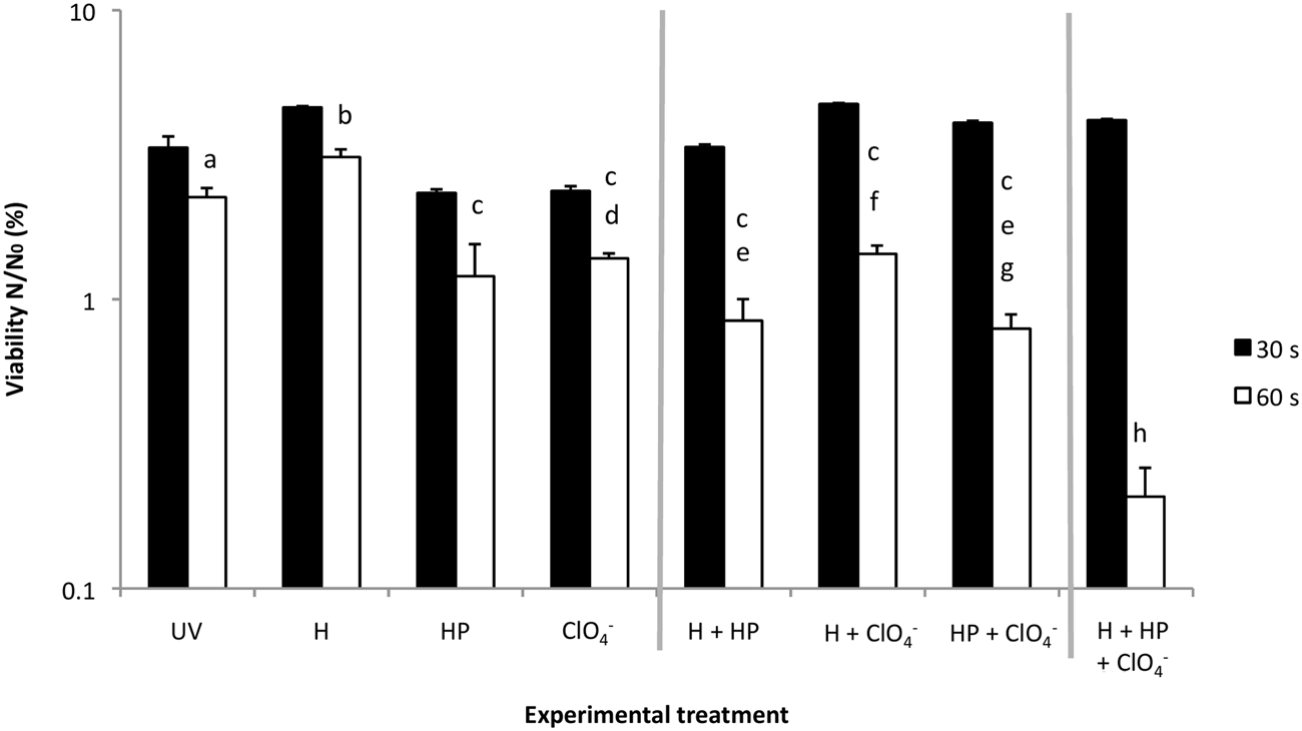
Perchlorate-induced bacteriocidal effects in the presence of other components of the Martian surface (hematite and hydrogen peroxide) after 30 and 60 seconds UV exposure. UV = UV irradiated control; H = 1 g/L hematite; HP = 10 mM hydrogen peroxide; ClO_4_ = 0.6 wt% Mg(ClO_4_)_2_. Letters shared in common between or among the groups indicate no significant difference (p > 0.05); error bars are + s.d. (n = 3). Vertical grey lines serve as visual separation of single, double and triple combinations.

We also examined the influence of two other components of the Martian surface environment: iron oxides and the oxidant hydrogen peroxide. We carried out experiments to determine whether these would act in synergy with irradiated perchlorates to make the surface of Mars inimical to life. Figure 5 shows the effects of the individual components, effects of combinations of two components and the combined effect of all three components under UV irradiation.

Experiments were conducted with the iron oxide, hematite, with a grain size of 5 μm (Sigma-Aldrich), which was added to the liquid system or rock analogue system at a concentration of 1 g/L. When hydrogen peroxide was used it was added to a final concentration of 10 mM. Mg(ClO_4_)_2_ was added to a concentration of 0.6 wt%, as in previous experiments.

Firstly, the individual components hematite, hydrogen peroxide and perchlorate were added to M9 containing *Bacillus subtilis* cells and irradiated under the monochromatic UVC source for the indicated length of time. UV-irradiated controls containing cells in M9 served as a control. Samples in the presence of hematite showed significantly higher cell viability after 60 seconds exposure than cells in the UV controls after the same length of time.

Samples individually treated with hydrogen peroxide or perchlorate showed a significant drop in viability in comparison to UV irradiated controls after 60 seconds.

Secondly, the individual components were paired as follows and irradiated for the indicated length of time: hematite and hydrogen peroxide; hematite and perchlorate; hydrogen peroxide and perchlorate. The combination of the iron oxide and hydrogen peroxide in the presence of UV radiation caused a significantly greater loss in viability than the individual components of hematite (3.7-fold) and perchlorate (1.6-fold). A decrease was also shown in comparison to samples treated only with irradiated hydrogen peroxide (1.4-fold). The iron oxide and perchlorate combination had a slight yet significant 1.04-fold increase in viability in comparison to perchlorate alone. Cells treated with perchlorate and hydrogen peroxide showed a 1.5-fold loss in viability, not significantly different to cells treated only with irradiated hydrogen peroxide; there was a significant 1.8-fold decrease in viability in comparison to samples just treated with irradiated perchlorate.

Lastly however, when combined, we found that all three components resulted in the largest drop in viability. After 60 seconds of UV radiation exposure cell viability was reduced to 0.21%, which was significantly lower than all other combinations examined.

## Discussion

Perchlorate, although stable at room temperature, is a powerful oxidant when activated, for instance at high temperatures^3^. Oxidants were hypothesized to be on the surface of Mars and responsible for the lack of organics found by the Viking missions^9, 10^. Perchlorate was not initially suspected as it was thought the majority of chlorine would occur as chloride ions^21^. Its presence was confirmed by NASA’s Phoenix Lander^1^ and recently spectrally identified as a component of brine seeps on the Martian surface by the Mars Reconnaissance Orbiter in late 2015^2^. Although perchlorate can be used as an electron acceptor for microbial growth, its potentially deleterious effects have been little explored.

We show that when magnesium perchlorate, at concentrations relevant to the Martian surface, is irradiated under short-wave UVC radiation encountered on the Martian surface it becomes bacteriocidal. We observe this effect both in liquid culture and in a rock analogue system that replicates a micro-environment within rocks. The effect is less pronounced within the rock analogue system likely caused by screening within the rock, which reduces the penetration of UV radiation compared to the liquid system. The bacteriocidal effect is also replicated when using other forms of perchlorate found in the Martian regolith: Calcium and sodium perchlorate. Both perchlorates significantly reduce viability of cells when irradiated in comparison to controls. Bacterial samples in the presence of perchlorate at Martian concentrations but in the absence of UV radiation show no loss of viability, which is consistent with the findings by Nicholson *et al*.^22^, that show no growth inhibition of *Bacillus subtilis 168* and *Bacillus pumilus* SAFR-032 when in the presence of perchlorate without UV exposure.

The mechanism of perchlorate action on cells is likely to be its degradation to deleterious reactive oxygen species. During irradiation, an increase in absorption at the expected maxima of hypochlorite (290 nm) and chlorite (260 nm) is observed. Similar photoproducts have been previously observed of perchlorate irradiated with ionizing radiation^3, 23^.

The chemical nature of this bacteriocidal effect is confirmed by carrying out the experiment at 4 °C, when the loss of viability is over ten times lower than at 25 °C, suggesting that lower temperatures lower the rate of the chemical reaction or the diffusion of activation products and reduce the rate of bacteriocidal effects. Nevertheless, the effect is still observable. The average surface temperature on Mars is approximately 218 K (−55 °C), however the Mars Exploration Rover Opportunity measured a daily maximum of 295 K (21.85 °C)^24^. Therefore, we would expect a range of reaction rates varying with latitude and time of day.

By lowering the perchlorate concentration by one order of magnitude to below that found at the Martian surface, the loss of viability is reduced to values not statistically significant from UV irradiation alone, showing that under conditions where perchlorates are diluted, the bacteriocidal effect is mitigated. By contrast, any environment that concentrates perchlorates, such as in putative Martian brines^13^, will produce uninhabitable environments on account of the bacteriocidal properties of irradiated perchlorates measured here. These properties suggest that the mere presence of liquid water seeps, thought to be good locations to search for life, does not imply environments fit for life.

Interactions of perchlorate with other major Martian soil components were investigated. Sulfate concentrations vary enormously across the Martian surface. Spectrometers onboard NASA’s Mars Explorations Rovers measured levels of sulfate at the Meridiani Planum with concentrations of 20–40 wt%^25^. We investigate the effects of sulfate at 30 wt% but see no effect on the perchlorate-induced loss of cell viability under UV irradiation.

However, other regolith components do have bacteriocidal properties. Mars has approximately 18 wt% iron oxides in their ferrous (Fe^2+^) and ferric (Fe^3+^) oxidation states combined^26^, which can participate in photochemical reactions.

A higher loss of cell viability is observed when hematite and hydrogen peroxide are combined than when they are added to cells individually under UV irradiation. These results can be explained by the Photo-Fenton reaction. The standard Fenton reaction consists of hydrogen peroxide reacting with catalytic ferrous iron, which results in its oxidation to ferric iron and the production of hydroxyl radicals^27^. The Photo-Fenton reaction is a more efficient variation as it utilizes UV light to catalyze the recycling of the iron (in dissolved or in oxide form)^28, 29^. The maximum bacteriocidal effect at neutral pH is achieved in the presence of 1 g/L iron oxide in an aqueous environment containing 10 mM hydrogen peroxide^30^.

When we combine hydrogen peroxide, hematite and perchlorates, which might represent a combination of compounds in the Martian soil, we observe the greatest loss of viability. We attribute this observation to the combined effect of UV-irradiated perchlorate-induced cell killing with Photo-Fenton-induced killing by iron oxides and hydrogen peroxides.

Although the toxic effects of oxidants on the Martian surface have been suspected for some time, our observations show that the surface of present-day Mars is highly deleterious to cells, caused by a toxic cocktail of oxidants, iron oxides, perchlorates and UV irradiation. There has been recent research into the use of perchlorates as a potential energy source for bacteria on Mars^6, 7^ and suggestions^31^ that such life may have been detected in the Viking Labeled Release experimental results. However, we show the bacteriocidal effects of UV-irradiated perchlorates provide yet further evidence that the surface of Mars is lethal to vegetative cells and renders much of the surface and near-surface regions uninhabitable. Our results show that even brine seeps, although they represent local regions of water availability, could be deleterious to cells, indigenous or contaminant if, as spectral evidence suggests, they contain perchlorates. The enhancement of the bacteriocidal properties of perchlorates by UV-irradiation suggest that these aqueous environments are even more deleterious to potential contaminants from spacecraft, and potentially less habitable, than was thought. These data have implications for planetary protection, specifically concerns about the forward contamination of Mars in both robotic and human exploration. Our work focuses on reporting the new finding of bacteriocidal properties of UV-irradiated perchlorate on life at ambient temperatures and under Martian conditions. While we present data on the underlying mechanistic possibilities (Supplementary Fig. S3), a study of the exact mechanism of damage and a review of affects on multiple bacteria species would constitute interesting lines of query for follow-up studies.

## Methods

### Bacterial preparation

Vegetative cells of *Bacillus subtilis* strain 168 (DM 402) were used in the experiment. This organism, although aerobic, is well-characterized model vegetative organism also found as a contaminant on spacecraft. To obtain aliquots for experiments, a monoculture was grown at 38 °C before being frozen into 25% glycerol 1 mL aliquots and stored at −80 °C.

To obtain overnight cultures for experiments, 200 mL nutrient broth was inoculated with thawed aliquots and cultured overnight at 38 °C before use in the experiments. The concentration of organisms in each culture was determined by serial dilution and plating on Oxoid (LP0011) nutrient agar.

In preparation for the radiation exposure experiments, 1 mL of the culture was centrifuged twice at 10,000 X g/min for 5 minutes and washed in between with Phosphate Buffered Saline (PBS). The pellet was resuspended in 1 mL Minimal Medium (M9) and transferred into a well of a 12-well plate. In this paper this is referred to as the ‘liquid system’. M9 media was used to rule out any secondary products being produced by irradiated organic molecules in the medium, which could additionally affect the cell viability. M9 contained the following: 10.5 g/L M9 salts (Sigma Aldrich) in distilled water; 2 mL of 1 M MgSO_4_; 10 mL of 20% glucose; 100 μL of 1 M CaCl_2_.

The bacterial concentration was kept below a maximum of 8 × 10^7^ cells/mL so that the theoretical surface area of the bacteria, even if they settled on the bottom of the well, would not exceed the surface area of the bottom of the well, causing self-shielding from multiple cell layers. As *Bacillus subtilis* is a motile bacterium and could move away from the iron on the well floor, multiple samples were taken with a pipette from the top half and the bottom half of the M9-bacteria suspension in the well after preparation. Cell concentration of the top and bottom samples was estimated using microscopy. Results show that samples taken from the bottom of the well contained 75% more bacteria than samples taken from the top half of the well. We therefore assumed that most of the bacteria were located on or very near to the well floor for the duration of the experiments and were most likely in close contact with the iron oxides. After irradiation, samples were always taken from the well floor.

### UV irradiation conditions

Mg(ClO_4_)_2_ hexahydrate (Sigma Aldrich) was added to obtain the final weight percentage of 0.6 wt%. If stated, hematite with a grain size of 5 μm (Sigma Aldrich) was added in to a final concentration of 1 g/L; hydrogen peroxide (30%) was added to the solution to a final concentration of 10 mM. This concentration of hydrogen peroxide was used as a decrease below 1 mM leads to inefficient Photo-Fenton catalyst function and an increase to 100 mM causes sterilization in the dark controls (Supplementary Fig. S5). Using 10 mM H_2_O_2_ also gave us the ability to compare our results to data published by Mammeri *et al*.^30^. The amount of 1 g/L iron oxide catalyst (when used) was chosen as UV light could still penetrate it and reach the cells; iron concentrations on Mars would vary substantially depending on location so this concentration was chosen as an example. Additionally, we could compare our data to that of Mammeri *et al*.^30^ as they tested a similar iron oxide catalyst concentration in heterogeneous Photo-Fenton reactions. Controls experiments were run using only bacteria in M9 medium. The 12-well plate was placed on a shaker for 1 minute to mix the components as homogenously as possible. For the experiments at 4 °C the plate was placed on ice for 30 minutes before and during irradiation, M9 media was stored at 4 °C prior to use.

UV irradiation experiments were carried out using two sources. In one set of experiments wells were irradiated with a monochromatic UVC lamp (λ = 254 nm; I = 11.2 W/m^2^) at distance of 5 cm for the indicated amount of time. This was performed aerobically and at 25 °C unless otherwise indicated. The effect of the monochromatic UVC was made comparable to Martian radiation by producing an absolute fluence which is similar to the fluence expected from the UVC to UVB regions (200–315 nm) on Mars^19^, albeit that biological effectiveness varies across the UV radiation spectrum. Nevertheless, as the biological effectiveness of processes such as DNA damage is similar across the short wavelength region, this monochromatic flux can be considered a reasonable proxy for short wavelength UV radiation damage.

To produce a more realistic UV radiation environment a polychromatic light was also used for irradiation and was generated by a 150 W Xe-Arc UV lamp (λ = 200–2500 nm; I = 0.010 W/(m^2^ nm)), which is part of the UK Centre for Astrobiology’s Mars chamber^32^. The spectral irradiance on the surface of Mars was calculated using the model from Cockell *et al*.^19^ and compared to the measured Xe lamp irradiance in the Mars chamber (Supplementary Fig. S2).

#### Rock analogue system

In order to recreate a more realistic environment for microorganisms on the surface of Mars, rock analogues were used to simulate the conditions for endolith (rock-dwelling) growth. These consisted of sintered discs of silica grains commercially produced to give a pore size of 100–160 µm (Scientific Glass, UK); they were 10 mm in diameter and 3 mm thick. The discs were soaked in the M9 medium and bacterial suspension, with the perchlorate already dissolved in the M9 medium before irradiation. This is referred to as the ‘rock analogue system’. After irradiation, the discs were gently crushed in a sterilized mortar and pestle in the presence of sterile water at given time points to extract the cells.

#### Anaerobic experiments

To test whether observed effects also occur under anaerobic conditions, some experiments were carried out in a Coy anaerobic chamber in a nitrogen atmosphere. For the anaerobic experiments, 10 mL M9 media was placed in a sealed, sterile serum bottle and degassed. This was achieved by purging the bottle with sterile, 0.22 μm-filtered N_2_ for 20 min/L using sterile needles to introduce the gas and relieve the pressure. After purging, the bottle was filled with 100% N_2_ headspace to maintain anaerobic conditions. The bacteria were then resuspended in the purged M9 in the anaerobic chamber and transferred into 12-well plates in which UV irradiation experiments were carried out as described in methods section *1*.

### Statistical analysis

Ten microlitre samples were taken from the wells/discs at given time points and plated on nutrient agar at a dilution of 1:100 and incubated overnight at 30 °C. Colonies were then counted and viability was expressed in terms of percentage of the starting cell concentration (N/N_0_ × 100%) where ‘N_0_’ = starting concentration and ‘N’ = surviving cells at the point of sampling. All experiments were performed in triplicate; numbers in figures show averages, error bars show standard deviation (s.d.) among triplicates. Non-irradiated, dark controls were run in all cases. Statistical analysis was performed using one-way ANOVA and two-tailed unpaired equal variance Students t-tests, where p < 0.05 was considered significant.

### Data availability

The authors declare the main data supporting the findings of this study are available within this article and its supplementary material.

## Electronic supplementary material

Supplementary material
