# Supplementary material for: Perchlorates on Mars enhance the bacteriocidal effects of UV light
Source: Sci Rep. 2017 Jul 6;7:4662. doi: 10.1038/s41598-017-04910-3 (PMC5500590; doi:10.1038/s41598-017-04910-3)
Supplement: Supplementary file 1 — Supplementary material [file 41598_2017_4910_MOESM1_ESM.pdf]

Supplementary material

# Perchlorates on Mars enhance the bacteriocidal effects of UV light

Jennifer Wadsworth[1*], Charles S Cockell[1]

[1]UK Centre for Astrobiology, School of Physics and Astronomy, University of Edinburgh, Edinburgh, EH10 4EP, UK

Email: j.l.wadsworth@sms.ed.ac.uk

Supplementary Fig. S1: Dark controls for M9 and perchlorate.

*Bacillus subtilis* in M9 medium, covered to control for light and sampled at given time points. M9 = cells in M9 medium; M9 + perchlorate = cells in M9 containing 0.6 wt% $Mg(ClO_4)_2$. Error bars are + s.d. (n = 3).

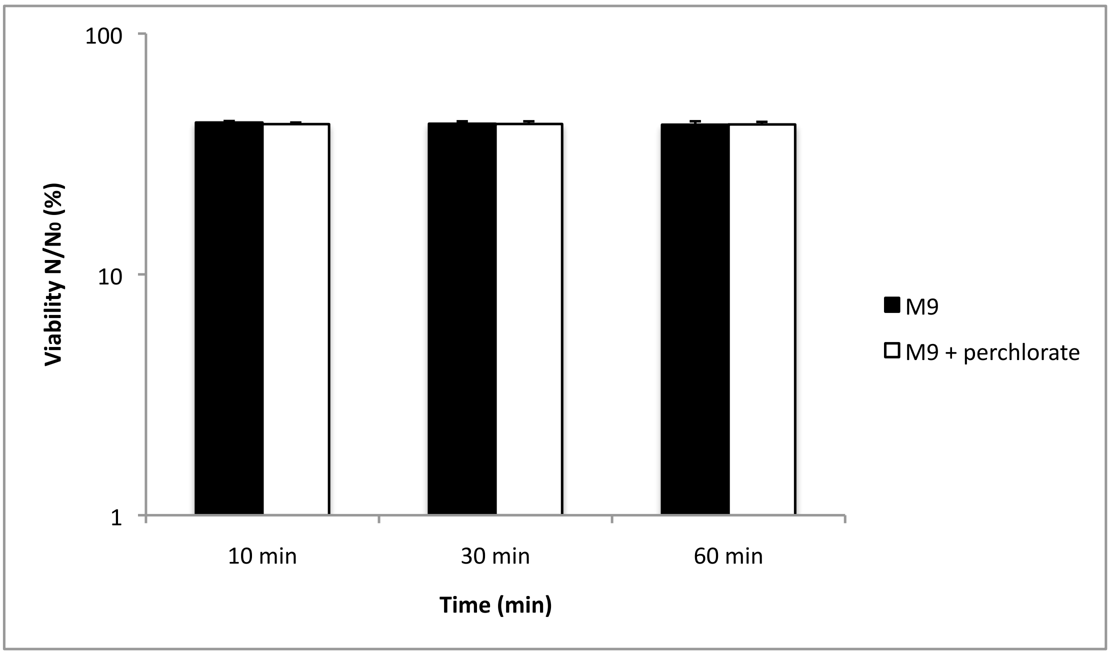

Supplementary Fig. S2: Modelled Mars UV irradiance vs. measured Mars chamber UV irradiance 200-400 nm.

Mars UV irradiance (mW/m$^2$/nm) was calculated using the model from Cockell et al.[1] with following parameters: OD = 1 ($\tau$ = 1.0); Sunlight angle 45°. Irradiance from polychromatic Xe lamp was measured in the Mars Chamber[2].

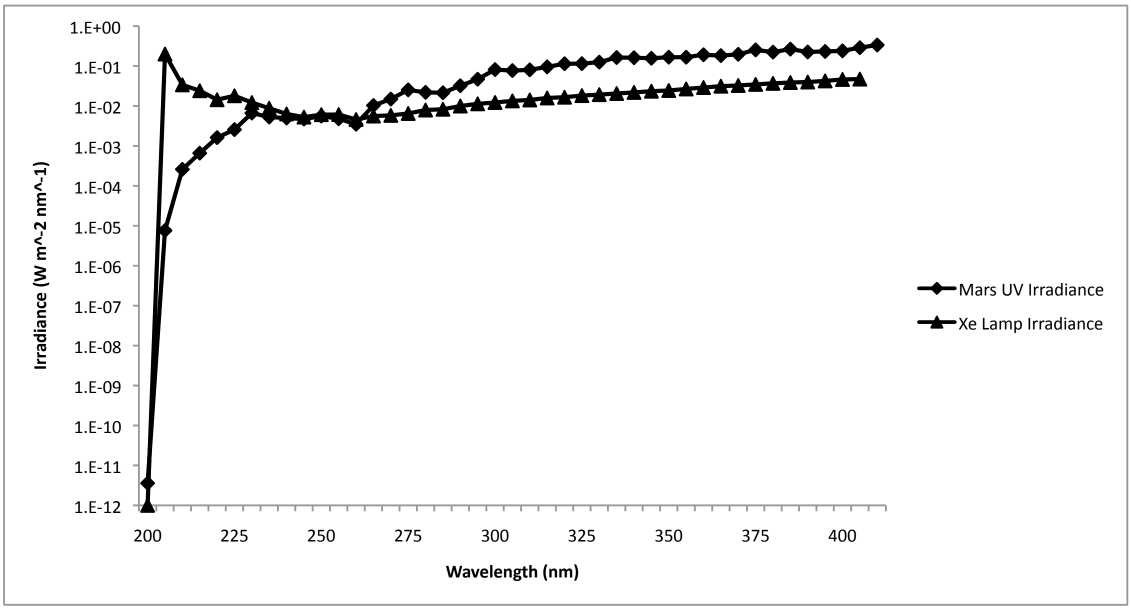

Supplementary Fig. S3: UV spectrophotometric absorbance of irradiated perchlorate: 6 wt% $Mg(ClO_4)_2$ irradiated with UVC.

UV-irradiated perchlorate (λ = 254nm) in sterile, distilled water; Absorbance at 260, 290 and 360 nm; Blanks used contained 6 wt% perchlorate.

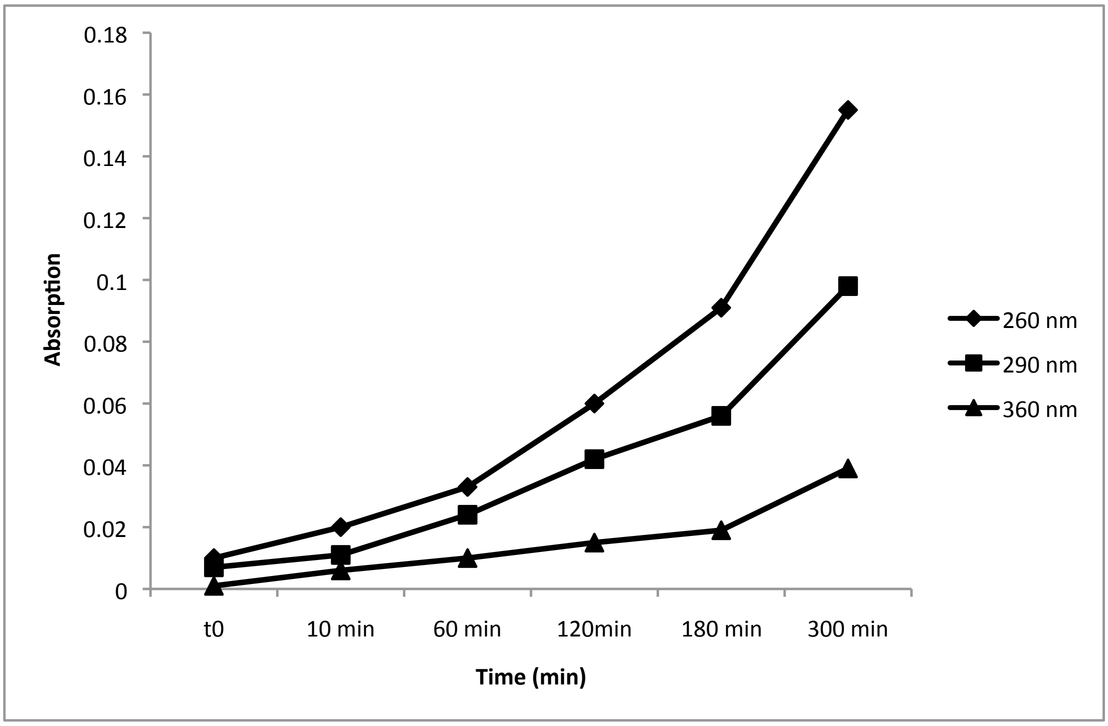

Supplementary Fig. S4: Effects of low concentrations of UVC-irradiated $Mg(ClO_4)_2$ on cell viability, 60 seconds exposure.

UV = UVC irradiated control; $ClO_4$- = $Mg(ClO_4)_2$ at given wt%.

$p < 0.05$ was considered statistically significant (*); error bars are + s.d. (n = 3).

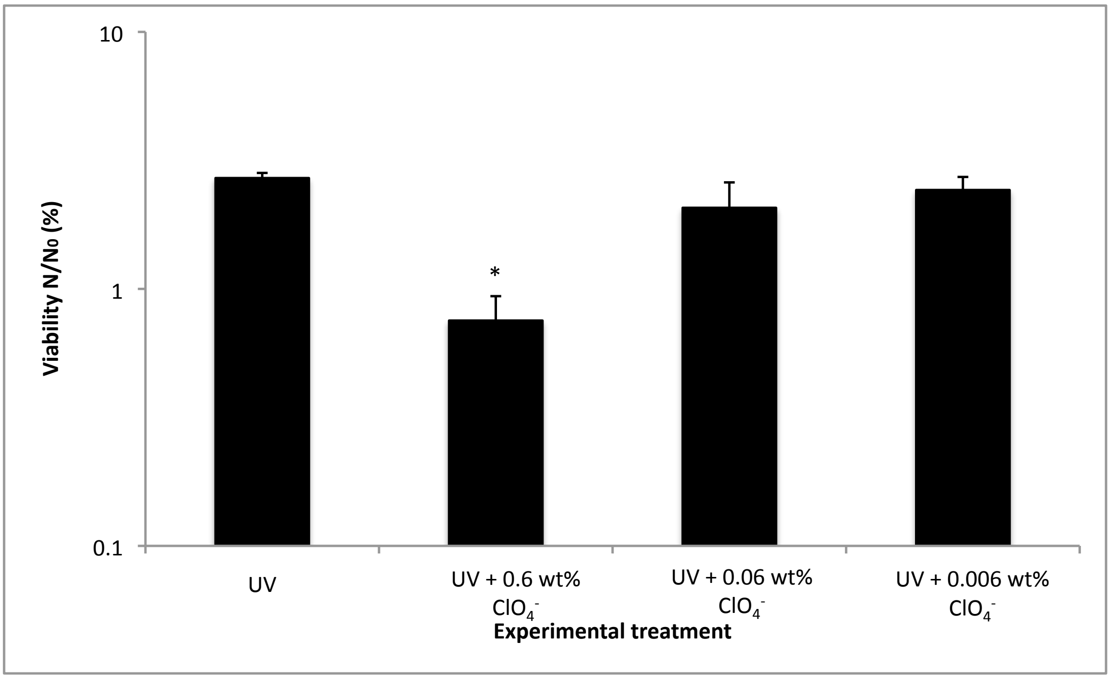

Supplementary Fig. S5: Effects of low and high concentrations of hydrogen peroxide on cell viability; irradiated and dark controls.

UV = irradiated with UV (λ = 254 nm); dark = covered samples; HP = hydrogen peroxide at given molarity. Error bars are + s.d. (n = 3).

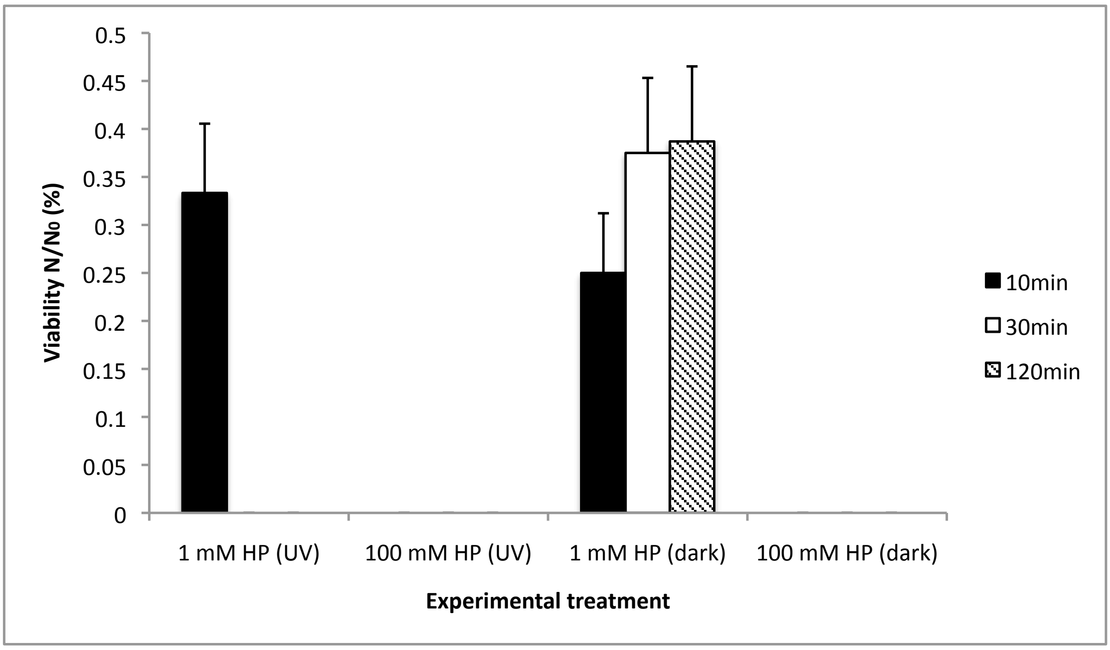

Supplementary Table S1: Summarized numerical results of viability of irradiated *B. subtilis* cells under various Martian analogue conditions.

Table contains numerical results from figures in the main paper. Results are in the unit $N/N_0$ [%], with 'N' as the number of surviving cells after a given treatment and '$N_0$' is the number of cells in the starting concentration calculated using the average of triplicates. The results for each condition are paired with the results from the respective UV-irradiated control ($\lambda$ = 254 nm, unless stated) and grouped into shaded or white rows. 'Mg-perchlorate' = $Mg(ClO_4)_2$ at given wt%; 'Ca-perchlorate' = $Ca(ClO_4)_2$ at given wt%; 'Na-perchlorate' = $NaClO_4$ at given wt%; Sulfate = $MgSO_4$ at given wt %; H = 1 g/L Hematite (5 μm grain size); HP = hydrogen peroxide at final concentration of 10 mM; polychromatic UV = 200-400 nm; n.a. = not applicable.

| | Irradiation time | |
|---|---|---|
| Conditions | 30 s | 60 s |
| UV control | 1.53 | 0 |
| UV + 0.6 wt% Mg-perchlorate | 0 | 0 |
| UV control | 45.86 | 22.22 |
| UV + 0.6 wt% Mg-perchlorate in rock analogue | 26.67 | 2.90 |
| UV control | n.a. | 0.12 |
| UV + 0.6 wt% Mg-perchlorate, anaerobic liquid system | n.a. | 0 |
| UV control | n.a. | 8.23 |
| UV + 0.6 wt% Mg-perchlorate, anaerobic rock analogue | n.a. | 0.85 |
| UV control | 0.36 | 0.15 |
| UV + 1 wt% Mg-perchlorate | 0.003 | 0 |
| UV + 2.5 wt% Mg-perchlorate | 0.003 | 0 |

| | | |
|---|---|---|
| UV + 5 wt% Mg-perchlorate | 0 | 0 |
| UV control | 3.93 | 0 |
| UV + 0.6 wt% Ca-perchlorate | 0 | 0 |
| UV + 0.6 wt% Na-perchlorate | 0.09 | 0 |
| UV control (16 x less irradiance) | 5.91 | 1.45 |
| UV + 0.6 wt% Ca-perchlorate (16 x less irradiance) | 7.36 | 0.54 |
| UV + 0.6 wt% Na-perchlorate (16 x less irradiance) | 6.07 | 0.69 |
| UV control | n.a. | 2.70 |
| UV + 30 wt% sulfate | n.a. | 2.41 |
| UV + 0.6 wt% Mg-perchlorate + 30 wt% sulfate | n.a. | 2.58 |
| UV control | 3.34 | 2.26 |
| UV + H | 4.61 | 3.10 |
| UV + HP | 2.33 | 1.20 |
| UV + 0.6 wt% Mg-perchlorate | 2.37 | 1.39 |
| UV + H + HP | 3.36 | 0.84 |
| UV + H + 0.6 wt% Mg-perchlorate | 4.74 | 1.44 |
| UV + HP + 0.6 wt% Mg-perchlorate | 4.08 | 0.79 |
| UV + H + HP + 0.6 wt% Mg-perchlorate | 4.16 | 0.21 |
| UV control | 1.70 | 1.27 |
| UV + 0.6 wt% Mg-perchlorate at 4°C | 3.32 | 1.69 |

| | 1 min | 2 min |
|---|---|---|
| UV control | 1.43 | 0.09 |

| | | |
|---|---|---|
| UV + 0.6 wt% Mg-perchlorate at 4°C | 1.31 | 0.008 |

| | 3 min | 4 min |
|---|---|---|
| UV control | 0 | 0 |
| UV + 0.6 wt% Mg-perchlorate at 4°C | 0 | 0 |

| | 10 s |
|---|---|
| Polychromatic UV control | 3.13 |
| Polychromatic UV + 0.6 wt% Mg-perchlorate | 0.29 |

Supplementary references

[1] Cockell. C. *et al.* The ultraviolet environment of Mars: Biological implications past, present, and future. *Icarus* **146,** 343-359 (2000).

[2] Martin D., Cockell C. S. PELS (Planetary Environmental Liquid Simulator): A new type of simulation facility to study extraterrestrial aqueous environments. *Astrobiology* **15,** 111-8 (2015).
